# The Emergence of Isotryptamine Analogs as Novel Serotonergic Agents with Psychotherapeutic Potential

**DOI:** 10.1021/acsptsci.6c00288

**Published:** 2026-08-18

**Authors:** Richard A. Glennon, Małgorzata Dukat

**Affiliations:** Department of Medicinal Chemistry, School of Pharmacy, 6889 Virginia Commonwealth University, Richmond, Virginia 23298, United States

**Keywords:** isoDMT, DMT, classical hallucinogens, psychedelics, 5-HT_2_ receptors, neuropsychiatric disorders

## Abstract

Isotryptamines, or 2-(1-indolyl)­ethylamines, are positional or geometric isomers of tryptamines where the aminoethyl substituent has been relocated from the indole 3-position to the N_1_-position. Early studies with these types of agents relied on peripheral tissue assays for examination of their serotonin (5-hydroxytryptamine; 5-HT) receptor affinities to investigate structure–affinity relationships. Once 5-HT receptors were identified in the brain and as the binding methodology improved, isotryptamines were further examined, including various *in vitro* and *in vivo* assays (i.e., model systems) as techniques evolved. Given that certain hallucinogenic/psychedelic tryptamines, such as analogs of *N,N*-dimethyltryptamine (DMT), are currently of interest in the treatment of various neuropsychiatric disorders, there is renewed interest in agents such as isoDMT, and an isoDMT analog has now entered clinical trials. The history of isotryptamine analogs, from their discovery to the present, is reviewed.

## Graphical abstract

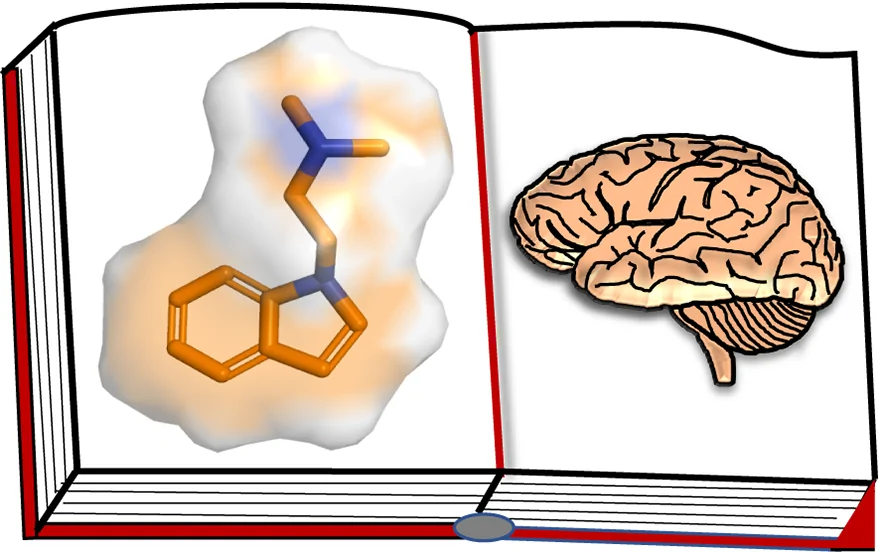

A very recent report indicates that there are more than 200 clinical trials ongoing with “psychedelics” to treat psychiatric disorders.^1^ In particular, there is growing interest in tryptamine-derived agents.^2^ Isotryptamine, or 2-(1-indolyl)­ethylamine (**1**), is a positional isomer of the naturally occurring trace amine tryptamine (**2**), just as *N*,*N*-dimethylisotryptamine (isoDMT; **3**) is a positional isomer of the naturally occurring hallucinogenic agent *N*,*N*-dimethyltryptamine (DMT; **4**) (Figure 1). The terms isotryptamine and isoDMT, as used here, were introduced in 1984.^3^

**Figure 1:**
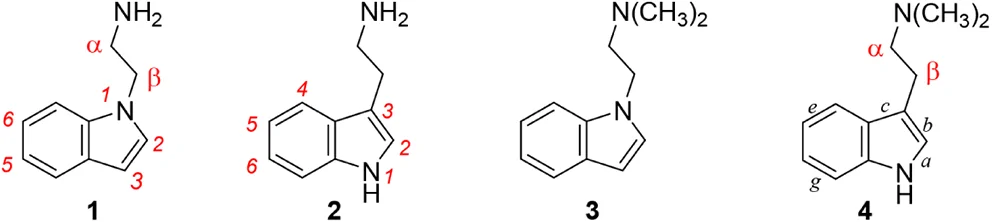
Structures/numbering of isotryptamine (**1**), tryptamine (**2**), isoDMT (**3**), and DMT (**4**).

DMT-related “*classical hallucinogens*” ^4–6^ (sometimes termed “*serotonergic hallucinogens*”) are also often referred to now as “*classical psychedelics*” or “*serotonergic psychedelics*”.^7^ Another term sometimes encountered is “*classic*” hallucinogen; as opposed to *classical* hallucinogen, the term *classic* hallucinogen was defined as an agent “*dependent upon the person who defines it and is an agent that achieves this status when it has served as the focus of a considerable amount of research attention*”.^8^ This is a rather vague and nonmechanistic definition. The terms “*classical*” hallucinogen and “*classical*” psychedelic might be synonymous but are not necessarily synonymous with their “*classic*” counterparts.^7^ Although the term “*hallucinogen*” has been the most widely used in the past medical literature to describe these agents,^9^ 30 years ago it was argued that it is a euphemism because hallucinations are not part of the usual syndrome.^8^ The term “*psychedelic*”, or mind manifesting, as first described in 1957 (and selected as a favorite from an array of new terms considered) by Osmond,^10^ might more accurately reflect the actions of these agents.^8^ A PubMed search (accessed May 1, 2026) for use of the terms “hallucinogen” (9,956 entries) and “psychedelic” (11,460 entries) for the period 2015–2025 indicates increased use of the latter in the past decade. Herein, the term “*hallucinogen*” refers to “*classical hallucinogens*” and the term “*psychedelic*” refers to “*serotonergic psychedelics*” and they will be used interchangeably. Agents such as, for example, ketamine, phencyclidine, and tetrahydrocannabinol are sometimes also referred to as psychedelic agents, and sometimes as psychotomimetics,^7,11^ but are not considered here as classical hallucinogens or serotonergic psychedelics.

DMT (**4**), one of the structurally simplest and better-investigated tryptamine-based classical hallucinogens, was first prepared, along with several other structurally related compounds, in 1931 by Manske.^12^ Investigation of DMT in human subjects began in the 1950s^13,14^ but was curtailed by the U.S. Controlled Substances Act of 1970. In the 1990s, Strassman and coworkers were the first to reinvestigate DMT in human subjects with government approval^15,16^ and published a detailed review of their extensive findings in 2001.^9^ Another DMT analog, psilocybin—the 4-phosphoryloxy analog of DMT—also saw renewed human investigation by Griffiths and colleagues, and related studies continue.^17,18^ Various tryptamine-related agents are currently being investigated for their psychotherapeutic potential (e.g., major depressive disorder, bipolar disorder, substance use disorders, neurodegenerative conditions).^19–25^ Clinical trials with DMT (**4**) have been reviewed,^2^ and a very recent Phase IIa clinical trial involving adults with moderate-to-severe major depressive disorder found that a single dose of DMT (**4**) produced a rapid and significant reduction in depressive symptoms that was sustained for up to 3 months.^2^ Another recent Phase IIa study investigated the antidepressant actions of vaporized DMT.^26^ On the basis of these findings, the present review will focus primarily, but not exclusively, on isotryptamines related to DMT (**4**).

The term “isotryptamine”, as used herein, refers to compounds where the 3-position aminoethyl chain of tryptamine (**2**) has been transposed to the indole N_1_-position [i.e., 2-(1-indolyl)­ethylamine; **1**; Figure 1].^3^ Given the interest in tryptamine analogs such as DMT (**4**) and some of its substituted and related derivatives, it would seem timely to visit the more obscure but structurally related isotryptamine positional isomers of the tryptamines. Despite the large number of known tryptamines, and particularly DMT (**4**) derivatives bearing various aryl substituents, alkyl modifications, and terminal amine groups, for which chemical and some pharmacological data are available,^8,27–29^ there still remain many isotryptamines to be explored. There are several general epochs in isotryptamine pharmacology: (i) early structure-affinity/structure–activity studies, (ii) subsequent follow-up studies including the synthesis and evaluation of novel isotryptamine-related compounds, and (iii) more recent psychopharmacological investigations that have advanced an isotryptamine to clinical trials. Nearly all pharmacological investigation of isotryptamine analogs has been based on studies using “*model systems*” that have some foundation in the actions of known hallucinogenic/psychedelic agents; the utility, applicability, reliability, and validity of certain of these models will be discussed. That is, the central actions of isotryptamines were being investigated at nearly the same time that various assay systems (e.g., radioligand binding, drug discrimination, head-twitch response) were evolving. Hence, codevelopments in some of these areas are required for data interpretation (or reinterpretation) so that the actions of tryptamines and their corresponding or related isotryptamine counterparts can be placed in proper context.

Although sparsely encountered in early scientific literature, the term “*isotryptamine*” probably first appeared in the title of a scientific article in 1969 by Snieckus and Bhandari^30^ to describe tryptamines (i.e., analogs of **2**) where the aminoethyl group was relocated from the indole 3-position to the 2-position [i.e., 2-(1*H*-2-indolyl)­ethylamines], not the N_1_-position. No pharmacological data were reported on these types of compounds until 1976, when Misztal et al.,^31^ described the analgesic effects of several amine-substituted analogs. Nevertheless, these 2-(1*H*-2-indolyl)­ethylamines have found application in the synthesis^32^ and pharmacological evaluation^33^ of tetrahydro-γ-carbolines and continue to be used in the synthesis of other related heterocyclic compounds^34^ or can be found embedded in them.^35^ The term isotryptamine is still occasionally employed to represent such compounds.^36^ However, to avoid any confusion, the present review will focus solely on isotryptamines related in structure to compound **1**, where the aminoethyl group is attached to the indole N_1_-position.

## Early Pharmacological Studies

Shortly following the discovery of serotonin (5-hydroxytryptamine; 5-HT), its actions were investigated using a variety of peripheral tissue preparations.^37^ One assay in particular that became widely used due to its sensitivity was the isolated rat stomach fundus preparation. Introduced in the late 1950s by Sir John Vane,^38^ the fundus preparation was used in pharmacological studies for many years to measure the affinity or “apparent affinity” of known and novel agents at fundus 5-HT receptors. Isolated tissue preparations could also be used to examine stereochemistry and the influence of an agent’s physicochemical properties on binding.^39^ DMT (**4**) was found to bind at these receptors, and its structure–affinity relationships were investigated.^40–44^ Although these receptors were not identified as being members of the 5-HT_2_ receptor family (i.e., 5-HT_2B_ receptors; *vide infra*) until decades later, this tissue preparation provided early qualitative and quantitative assessments of drug-receptor interactions at 5-HT receptors for quite some time. In an attempt to identify structural features that contributed to the binding of DMT (**4**), various DMT-related agents were examined;^3,45^ a review of the topic with specific data on a number of DMT analogs and related agents has been published,^46^ but for the sake of simplicity, results will only be discussed here in general terms. One structure-affinity study investigated the role of the indole N_1_-nitrogen atom and C_2_=C_3_ unsaturation (Figure 2); the indene counterpart of DMT (i.e., **5**, also known as C-DMT; originally studied by Winter et al.^42^) displayed half the affinity of DMT (**4**).^45^ Likewise, although binding with lower affinity than DMT (**4**), indane **7** displayed half the affinity of indoline **6**. It might be noted that indoline **6** and indane **7** possess a chiral center and exist as a pair of optical isomers; the individual isomers were not examined. However, the racemate and both optical isomers of the N-*des*-monomethyl counterpart of **7** were evaluated, and the (+)-isomer possessed twice the affinity of its racemate and was comparable in affinity to racemic **6**;^45^ hence, stereochemistry seemed to play a small role in binding.

**Figure 2:**
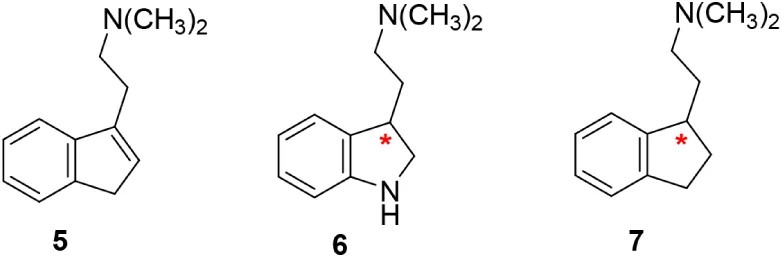
Indene, indoline, and indane analogs **5**–**7**, respectively, of DMT (**4**). Asterisk denotes chiral center.

Excision of the 5-membered indole ring of DMT (see Figure 3) to afford the 3-(phenyl)­propylamine **8** resulted in decreased affinity, suggesting that either the ring nitrogen atom and/or the C_2_=C_3_ double bond might be required for optimal binding. However, the binding of indene **5** and indane **7** would argue that these features are not strict requirements for binding, because they displayed, albeit somewhat reduced, affinity. Nevertheless, replacement of the benzylic methylene group of **8** by a nitrogen atom (i.e., *N*-aminoethylaniline **9**) resulted in a 10-fold enhanced affinity and in a compound with an affinity nearly comparable to that of DMT (**4**);^3,45^ here, it was speculated that the electronic character of the anilino nitrogen atom of **9** might be mimicking that of the indolic C_3_ atom of DMT (**4**).

**Figure 3:**
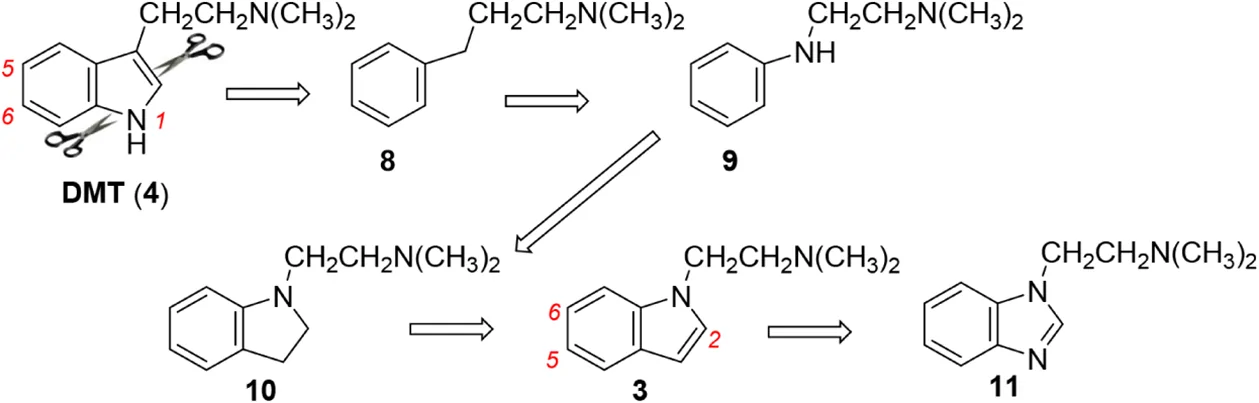
Structural evolution of isotryptamine analogs of DMT (**4**).

Another consideration relates to conformational flexibility. Conversion of **8** to a conformationally constrained molecule (*viz*. **5** and **7**) resulted in enhanced affinity, as did conversion of **9** to **10**. Introduction of unsaturation to **10** to afford isotryptamine **3** (termed isoDMT^3^) provided further enhancement of affinity such that **3** displayed nearly 10-fold higher affinity than DMT (**4**). Reintroduction of the N_1_-indolic nitrogen atom of DMT to isoDMT, to afford the benzimidazole analog **11**, had little effect on affinity. For a comparison of relative affinities: phenylpropylamine **8** < indane **7** < indoline **6** ≈ indene **5** < aminoethylaniline **9** ≈ DMT (**4**) < benzimidazole **11** < isoDMT (**3**). Later, aryl-substituted DMT analogs were examined, and affinity varied in the following order (shown here for some representative examples): 5-methoxy DMT > psilocin (i.e., 4-OH DMT) > 7-Br DMT > 4-methoxy DMT > DMT > 6-methoxy DMT > 7-methoxy DMT.^46^

Subsequent studies that examined isotryptamines focused primarily on analogs of isoDMT because homologation of the *N*,*N*-dimethyl group of isoDMT (**3**) to *N*,*N*-diethyl or *N*,*N*-di-*n*-propyl groups (i.e., isoDET and isoDPT, respectively) resulted in somewhat decreased affinity.^46^ With respect to ring-substituted isoDMT analogs,^46^ 6-methoxy isoDMT (**12**) possessed about 5-fold higher affinity than its corresponding DMT counterpart (i.e., 5-methoxy DMT; **13**) whereas the affinity of 5-methoxy isoDMT (**14**) was 10-fold lower than that of **13** (Figure 4). The affinity of **15** was 20-fold lower than that of **13**. As time went on, there was growing evidence that 6-methoxy isoDMT (**12**) behaved in a manner akin to 5-methoxy DMT (**13**) and that 5-methoxy isoDMT (**14**) behaved more like 6-methoxy DMT (**15**). That is, it seemed that isotryptamines and tryptamines were not binding in such a manner that their indole nuclei were strictly superimposed, but that tryptamines and isotryptamines behaved as isosteres or bioisosteres when the C_3_-substituent of the tryptamines was moved to the N_1_-position.^3,44^

**Figure 4:**
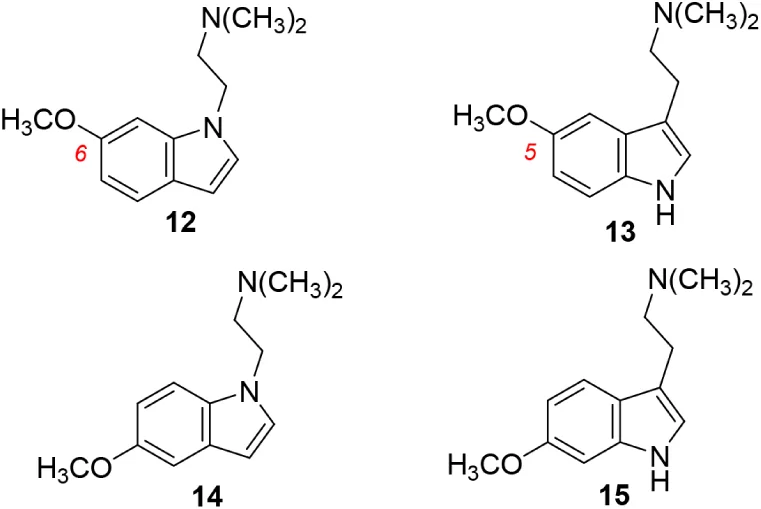
6-methoxy isoDMT (**12**), 5-methoxy DMT (**13**), 5-methoxy isoDMT (**14**), and 6-methoxy DMT (**15**).

To test this “indolic overlay” concept, a second *N*,*N*-dimethylaminoethyl group was added to the 3-position of isoDMT (**3**), corresponding to the N_1_-position of DMT (**4**), to afford a 3,4-disubstituted hybrid (Figure 5). The concept was that tryptamines might accommodate two dimethylaminoethyl groups, one at the N_1_-position and another at the C_3_-position. As it turned out, the “**3**/**4 hybrid**” compound behaved in a noncompetitive manner in the fundus assay, and its affinity could not be determined.^3^

**Figure 5:**
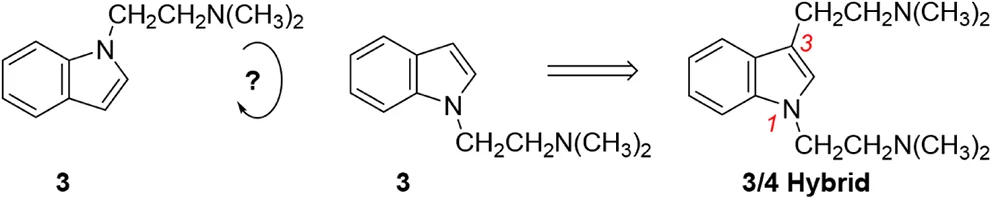
To test the hypothesis that isotryptamines might or might not bind in an isosteric fashion with their DMT (**4**) counterparts, a DMT (**4**)/isoDMT (**3**) hybrid analog was synthesized with dimethylaminoethyl substituents at both the N_1_ and C_3_ positions.^3^

Various phenylalkylamine hallucinogens also bind at fundus 5-HT receptors, including the phenylethylamine mescaline and phenylisopropylamines, or 1-(4-X-2,5-dimethoxyphenyl)-2-aminopropanes such as DOM (**16**) and DOB (**17**), where X is methyl and bromo, respectively; **16** and **17** (Figure 6) displayed higher affinity than DMT (**4**).^46,47^ DOI (**18**), where X = iodo, was subsequently found to bind as well, and for these types of compounds, the *R*(−)-isomers represented their eutomers.^48,49^ The structure–affinity relationships of 45 phenylalkylamines were examined.^50^ In addition, there was a significant correlation between their affinity for rat fundus receptors and the hallucinogenic potency of 14 agents for which human data were then available.^48^ This was the first evidence suggesting that phenylalkylamine hallucinogens, as a group, might involve a serotonergic mechanism. These types of agents are further mentioned below.

**Figure 6:**
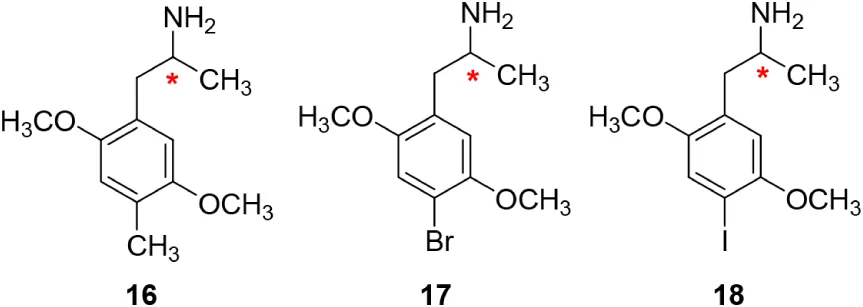
Phenylisopropylamines DOM (**16**), DOB (**17**), and DOI (**18**) showing chiral centers (denoted by asterisks).

## Radioligand Binding Studies

Most investigators gradually abandoned use of peripheral tissue assays to examine serotonergic agents once radioligand binding techniques using brain tissue homogenates were introduced. Tritiated 5-HT labeled a population of rat brain sites for which isotryptamines displayed low affinity; for example, the affinity of 6-methoxy isoDMT (**12**) was 10-fold lower than that of 5-methoxy DMT (**13**).^3^ Following several years of investigation by various laboratories, two different populations of 5-HT receptors were identified in the brain: 5-HT_1_ and 5-HT_2_.^51^ Early studies were based on binding data from these two receptor populations; later, 5-HT_1A_ and 5-HT_1B_ receptors were identified, leading to confusion interpreting 5-HT_1_ binding results. Continued investigation and the introduction of new radioligands eventually identified seven families of 5-HT receptors, 5-HT_1_–5-HT_7_ receptors, and several were found to consist of multiple subpopulations (e.g., 5-HT_1A_, 5-HT_1B_, 5-HT_1D_, 5-HT_1E_, 5-HT_1F_, 5-HT_2A_–5-HT_2C_) (reviewed by Barnes et al.^52^). Later, rat fundus receptors were cloned, initially termed 5-HT_2F_ receptors,^53,54^ and very shortly thereafter renamed 5-HT_2B_ receptors.^52^ Early brain binding studies used different techniques (e.g., radioligands, animal species, and/or tissue sources); hence, the most reliable affinity comparisons are likely derived from comparative data within a specifically cited paper rather than between papers from different laboratories. And, even then, techniques in a given laboratory evolved as time progressed (e.g., from rat brain tissue, human brain tissue, cloned rat receptors, and cloned human receptors to newer radioligands). Here, where relevant and available, radioligand and tissue source (e.g., rat versus human) will be cited. Also, with respect to 5-HT_2_ receptor affinity measurements, the most common radioligand today is probably the antagonist [^3^H]­ketanserin. Radiolabeled 5-HT_2_ receptor agonists such as tritiated DOB (**17**) or radioiodinated DOI (**18**) (that is, e.g., [^3^H]­DOB^55^ and [^125^I]­DOI^56,57^ and the individual radioiodinated optical isomers of DOI^58^) were introduced to label the high-affinity state of these receptors. 5-HT_2_ receptor agonists displayed up to, and sometimes greater than, 100-fold higher affinity for radiolabeled agonists versus [^3^H]­ketanserin-labeled 5-HT_2A_ receptors.^56^

Once 5-HT_2_ (i.e., 5-HT_2A_ and 5-HT_2C_ and later 5-HT_2B_) receptors were identified in the brain, several isotryptamines were examined at rat and human 5-HT_2A_ receptors.^59^ Supporting the claim derived from rat fundus studies, DMT and isoDMT analogs did not seem to bind at 5-HT_2A_ receptors in a manner where their indolic nuclei were strictly superimposed. That is, the affinity of 5-methoxy DMT (**13**; *K*_i_ = 600 nM at [^3^H]­ketanserin-labeled r5-HT_2A_ receptors) was higher than that of 6-methoxy DMT (**15**; *K*_i_ = 7,300 nM), whereas that of 5-methoxy isoDMT (**14**; *K*_i_ > 10,000 nM) was substantially lower than that of 6-methoxy isoDMT (**12**; *K*_i_ = 1,040 nM).^60^

Because α-methyltryptamine (AMT; **19a**) (Figure 7), a recognized hallucinogenic agent^61^ with reportedly three times the potency of DMT in human subjects^8^ (although routes of administration were different), (i) binds at rat fundus 5-HT receptors with similar affinity as tryptamine (**2**), and with the affinity of *S*(+)­AMT twice that of racemic AMT and nearly ten times that of *R*(−)­AMT,^62^ (ii) binds at [^3^H]­ketanserin-labeled r5-HT_2_ receptors (*K*_i_ = 2,500 nM) with half the affinity of DMT (**4**), and with the affinity of *S*(+)­AMT nearly twice that of *R*(−)­AMT,^60^ and because (iii) *S*(+)­5-methoxy AMT binds with nearly 10-fold higher affinity than *R*(−)­AMT,^60^ isoAMT (**20**) was examined and found to bind at [^3^H]­ketanserin-labeled rat brain 5-HT_2A_ receptors with half the affinity of AMT (**19a**).^60^ Introduction of certain aryl substituents enhanced the affinity of isoAMT, prompting examination of additional compounds. Among the compounds examined at [^3^H]­ketanserin-labeled r5-HT_2A_ receptors were **21** and **22** (Figure 7); although they bind (*K*_i_ = 640 and 320 nM, respectively), their affinity was higher when [^3^H]­DOB was used as the radioligand (*K*_i_ = 230 and 15 nM, respectively).^59^ The higher affinity for agonist-radiolabeled receptors suggested that **22** might be a 5-HT_2A_ receptor agonist. Compound **22** also displayed high affinity (*K*_i_ = 39 nM) for [^3^H]­DOB-labeled human 5-HT_2A_ receptors.^59^ Bös et al.,^63^ examined a series of α-alkyl isotryptamines with methoxy, alkyl, and halo substituents at various aryl positions. In certain cases, individual optical isomers were examined and were found to display minimal (2- to 4-fold) differences in affinity. Both the difluoro analog **22** and its opposite enantiomer bind at [^3^H]­DOB-labeled human 5-HT_2A_ receptors with identical affinity (*K*_i_ = 100 nM), and both were nearly equipotent and equi-efficacious as full agonists in a functional (IP_3_ accumulation) assay.^63^ Although some of the isotryptamines displayed up to 125-fold selectivity for 5-HT_2C_ versus 5-HT_2A_ receptors, this could not be replicated;^59^ nevertheless, selectivity might be the result of particular aryl-substitution patterns.

**Figure 7:**
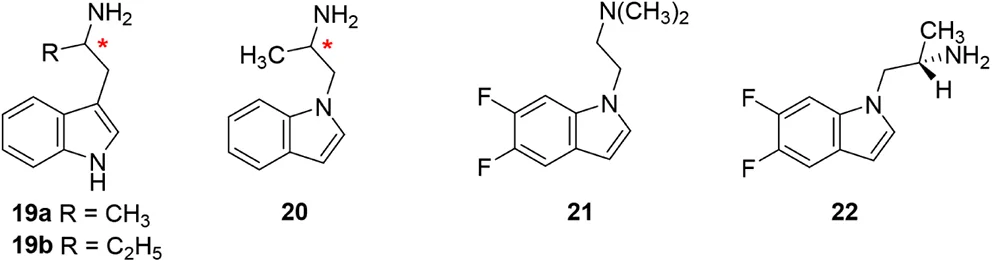
α-methyltryptamine (AMT; **19a**), α-ethyltryptamine (AET; **19b**), 5,6-difluoro isoDMT (**21**), and *S*-5,6-difluoro isoAMT (**22**). Chiral centers are identified and denoted by asterisks.

In addition to binding at antagonist-labeled r5-HT_2_ receptors,^60^ a subsequent investigation using agonist-labeled human 5-HT_2_ receptors revealed high affinity for AMT (**19a**) at 5-HT_2A_ receptors (*K*_i_ = 16 nM with [^125^I]­DOI as radioligand) and somewhat higher affinity at 5-HT_2B_ and 5-HT_2C_ receptors (*K*_i_ = 6.9 and 2.7 nM, respectively, with [^3^H]­5-HT as radioligand).^64^ For comparison, the 5-HT_2_ agonist DOB (**17**) displayed *K*_i_ values of 1.2 nM, 44 nM, and 6.3 nM, respectively, for these three receptor populations.^64^ AMT (**19a**) is also a releasing agent at monoamine transporters (i.e., serotonin, dopamine, and norepinephrine transporters, or SERT, DAT, and NET, respectively)^65^ (Table 1). The (−)-isomer of isoAMT (**20**) was also a monoamine releasing agent with a slightly different potency/selectivity profile (Table 1). Abuse-related effects of AMT (**19a**) and the isoAMT optical isomer were evaluated in assays of intracranial self-stimulation (ICSS) in rats and cocaine discrimination in rats and rhesus monkeys; neither reliably facilitated ICSS nor substituted for cocaine.^65^ In a separate investigation^66^ AMT (**19a**) was compared with its homologue, α-ethyltryptamine (AET; **19b**) (Figure 7), using rat brain synaptosomal homogenates (Table 1). Additionally, AMT (**19a**) was shown to be a full agonist (EC_50_ = 23.1 nM) in a 5-HT_2A_ Ca^2+^ mobilization assay.^66^

**Table 1:** Releasing Potencies of AMT (**19a**), isoAMT, and AET (**19b**) at Monoamine Transporters^65,66^

	EC_50_ (nM)		
	SERT	DAT	NET
(±)AMT(**19a**)^65^	28	173	79
(−)-isoAMT	177	1,062	81
(±)AMT(**19a)** ^66^	21.7	78.6	112
(±)AET (**19b**)	23.2	232	640
*S*(+)AET	34.7	57.6	592
*R*(−)AET	54.9	654	3,670

Serotonin binds with high affinity at 5-HT_1E_ (*K*_i_ = 10 nM) and 5-HT_1F_ (*K*_i_ = 12 nM) receptors; however, DMT (**4**) binds with reduced affinity (*K*_i_ = 300 and 130 nM, respectively).^67,68^ Several dozen tryptamine analogs were examined at these receptors, and 5-methoxy isoDMT (**14**) (*K*_i_ > 10,000 and 2,265 nM for 5-HT_1E_ and 5-HT_1F_ receptors, respectively) displayed low affinity.^67,68^ Isotryptamines also bind at 5-HT_6_ receptors; however, these typically bear a substituent at the indole 3-position, such as **23** (*K*_i_ = 20 nM), **24** (*K*_i_ = 22 nM), and **25** (*K*_i_ = 32 nM) (Figure 8); for comparison, the N_1_-benzenesulfonyl analog and N_1_-benzyl analog of DMT bind with *K*_i_ values of 4.1 and 6.0 nM, respectively.^69,70^ The *R*(−)-isomer of compound **26** (*K*_i_ = 0.9 nM) was found to bind at 5-HT_6_ receptors with 30-fold higher affinity than its *S*(+) antipode (*K*_i_ = 29 nM).^71^

**Figure 8:**
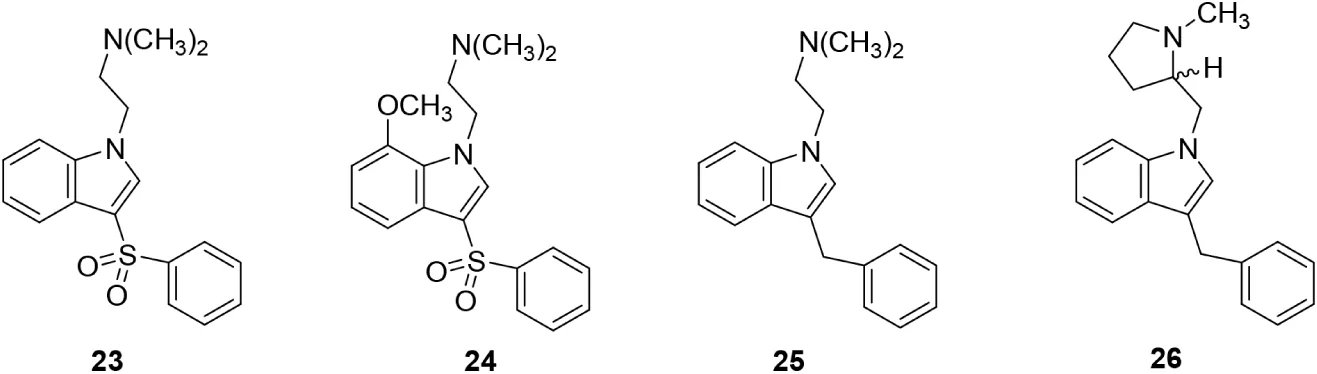
3-position substituted isoDMT analogs with affinity for 5-HT_6_ receptors.

On the basis of early molecular modeling studies (that would be considered quite rudimentary by today’s standards), it was proposed that 5-HT_2A_ (and perhaps 5-HT_1A_) receptors might possess a region of bulk tolerance associated with the *e*-face of tryptamines^72^ [for *face* names, see structure of DMT (**4**) in Figure 1]. Consequently, benz­[*e*]-fused analogs of DMT (**4**), such as **27**, were prepared and examined (Figure 9); the benz­[*g*]-fused analog of DMT (i.e., **28**) was employed as a comparator. The benz­[*f*]-fused **29** remains to be investigated. Indeed, benz­[*e*]­DMT (**27**) displayed higher affinity than DMT (**4**) or benz­[*g*]­DMT (**28**) at [^3^H]­ketanserin-labeled r5-HT_2A_ receptors (Table 2).^73^ Because 5-HT_2A_ receptor agonists display higher affinity for 5-HT_2A_ receptors labeled by an agonist radioligand than when [^3^H]­ketanserin is employed, these compounds were also examined using [^3^H]­DOB as a radioligand (Table 2). Compound **27** displayed about 20-fold higher affinity than DMT (**4**) for the latter.

**Figure 9:**
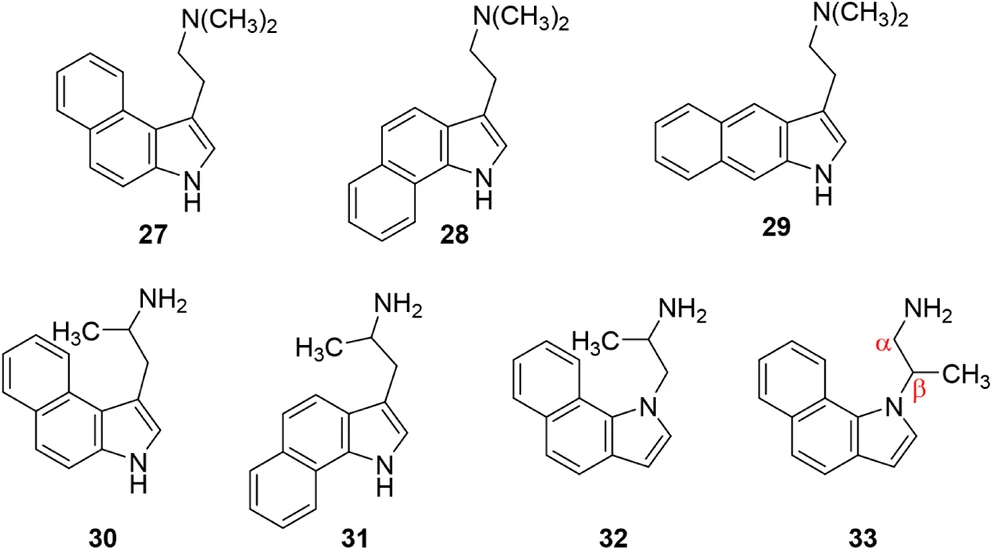
Benz-fused analogs of DMT (i.e., **27**–**29**), AMT (**30**, **31**), and isoAMT (**32**, **33**).

**Table 2:** Selected 5-HT Receptor Binding Data for Some Benz-Fused Tryptamines as Compared to DMT (**4**)^73^

	*K*_i_ (nM)				
	DMT (4)	Benz[*e*]DMT (**27**)	Benz[*g*]DMT (**28**)	Benz[*e*]AMT (**30**)	Benz[*g*]AMT (**31**)
**5-HT**_**1A**_	160	28	3,900	690	>10,000
**5-HT**_**1D**_	113	8	3,780	2,720	>10,000
**5-HT**_2A(K)_ ^a^	975	71	285	285	2,880
**5-HT**_2A(D)_ ^a^	56	3	26	3	125
**5-HT**_**2C**_	260	22	310	9	2,550
**5-HT**_**3**_	-	-	-	>10,000	>10,000

a K indicates that [^3^H]­ketanserin was used as radioligand, whereas D indicates [^3^H]­DOB was used.

Benz-fused analogs of AMT (**19a**) also were examined at 5-HT_2A_ receptors.^74^ Here, too, racemic benz­[*e*]­AMT (**30**) displayed higher affinity for this receptor population than benz­[*g*]­AMT (**31**) (Table 2). The similarly fused isotryptamine **32** (*K*_i_ = 38 nM) displayed 74-fold enhanced 5-HT_2A_ receptor affinity compared to its benz­[*g*]-fused AMT (**31**; *K*_i_ = 2,880 nM) counterpart. Relocation of the α-methyl group of **32** to the β-position (i.e., **33**; *K*_i_ = 4,300 nM) resulted in >100-fold decreased 5-HT_2A_ receptor affinity.^59^ Hence, from the compounds examined, benz-fused isotryptamine analogs of DMT and AMT seemed to act at 5-HT_2A_ and 5-HT_2C_ receptors in a similar isosteric manner as their DMT and AMT counterparts. These compounds were not examined at 5-HT_2B_ receptors.

### Conformationally Constrained Isotryptamines

Compound **26** (*vide supra*) is an example of a conformationally constrained analog of isoDMT (**3**). Another type of conformational constraint tethers the aminoethyl chain to the isotryptamine 2-position; pyrazino­[1,2-*a*]­indoles such as **34** and **35** are examples of this (Figure 10). The constrained isoDMT analog **34a** displayed 5-HT_2A_ receptor affinity comparable to isoDMT (**3**), but lower affinity than DMT for 5-HT_2C_ receptors (Table 3). Affinity appeared to be substituent-dependent; compounds **34f** and **35a** displayed higher 5-HT_2A_ receptor affinity than isoDMT (**3**), whereas those of **34c** and **34d** were lower, indicating that the affinity of pyrazino­[1,2-*a*]­indoles is susceptible to structural modification. It might be mentioned, *en passant*, that **34** represents the isotryptamine counterpart of the tryptamine scaffold embedded in the naturally occurring hallucinogenic β-carboline harmala alkaloids (i.e., **36**) (Figure 10) whose 5-HT_2A_ (and other) receptor binding properties have been examined.^75,76^ On the basis of earlier rat fundus binding data and the constrained benzimidazole analog of **11** (i.e., **37**) that reintroduces the indolic nitrogen atom common to some harmala alkaloids that bind with high 5-HT_2A_ receptor affinity,^75^ it was unexpected that **37** would display such low affinity.

**Table 3:** Affinity of Constrained Isotryptamines for [^3^H]­ketanserin (5-HT_2A(K)_)- and [^3^H]­DOB (5-HT_2A(D)_)-Labeled, and [^3^H]­mesulergine (5-HT_2C_)-Labeled Rat Brain 5-HT_2_ Receptors,^59^ as Well as for I_2_ Imidazoline Receptors and α_2_-adrenoceptors (α_2_-ARs)^77^ Using Rat Brain Homogenates as Previously Described^78^

		*K*_i_ (nM)				
		5-HT_1A(K)_	5-HT_2A(D)_	5-HT_2C_	I_2_	α_2_-AR
**34a**	H^a^	1,040	635	4,780	6.5	516
**34b**	6-OCH_3_	1,000	300	1,990	-	-
**34c**	7-OCH_3_	5,190	1,300	5,160	250	4,510
**34d**	8-OCH_3_	5,830	930	9,930	6.2	9,550
**34e**	6-Cl	630	330	520	-	-
**34f**	7-Cl	58	80	560	-	-
**34g**	8-Cl	1,670	320	1,460	-	-
**35a**	-	130	60	330	-	-
**35b**	-	1,250		450	-	-
**37**	-	>10,000	>10,000	>10,000	-	-

a For comparison, isoDMT (**3**) *K*_i_ values are 600, 650, and 720 nM, respectively, for the three 5-HT receptor columns identified above, and the affinity of the 5- and 6-methoxy analogs of isoDMT (i.e., **14** and **12**, respectively) at 5-HT_2A(K)_ receptors are >10,000 nM and 1,040 nM, and at 5-HT_2C_ receptors are >10,000 nM and 490 nM, respectively.^59^

**Figure 10:**
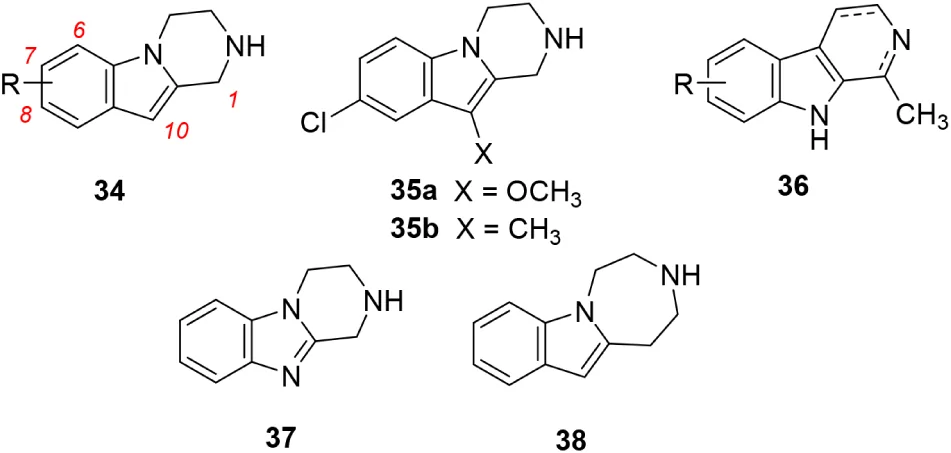
Examples of constrained isotryptamine analogs **34**, **35**, **37**, and **38**, and the general structure of harmala alkaloids (**36**).

There was a time when there were few I_2_ imidazoline receptor ligands available that did not bind at I_1_ receptors and/or α_2_-adrenoceptors (α_2_-ARs). Interestingly, pyrazino­[1,2-*a*]­indoles **34a** and **34d** represented some of the first compounds with I_2_ selectivity (Table 3). In particular, **34d** (8-OMe THPI; *K*_i_ = 6.2 nM) binds at rat brain I_2_ receptors with >1000-selectivity over α_2_-ARs (Table 3) and I_1_ receptors (IC_50_ = 8,280 nM).^77^ The chemistry and pharmacology of pyrazino­[1,2-*a*]­indoles have been reviewed by Zhang et al.^79^

Ennis et al.^80,81^ described a large number of substituted derivatives of isotryptamine-related azepinoindoles (**38**), ring-expanded analogs of **34**, that bind at 5-HT_2A_ and 5-HT_2C_ receptors with high affinity and varying selectivity for the two 5-HT_2_ receptor subtypes; compound **38** (PNU-181731) itself binds with *K*_i_ values of 18 and 4.8 nM, respectively. Several analogs demonstrated anxiolytic action in a mouse model.^80^

The hallucinogenic/psychedelic agent lysergic acid diethylamide (LSD; **39**) (Figure 11) is a nonselective agent that binds at 5-HT_2A_ receptors with low- to subnanomolar affinity. Recently, a series of LSD-related isotryptamine analogs **40** has been reported; although the ring numbering system differs for **39** and **40**, that of the better-known LSD (**39**) will be used here to make comparisons. Note also that LSD has two different ring numbering systems: the ergoline system (the system most commonly employed and that used here) and the IUPAC system. The IUPAC name for LSD is (6a*R*,9*R*)-*N*,*N*-diethyl-7-methyl-4,5,6a,7,8,9-hexahydroindolo­[4,3-*fg*]­quinoline-9-carboxamide; hence, the two stereocenters, using IUPAC nomenclature, would be at the 6a- and 9-positions rather than at the 5- and 8-positions. Tetracyclic isotryptamines bearing a chloro or fluoro substituent on the LSD A-ring (i.e., the LSD 12-, 13-, or 14-position), and with varying stereochemistry at the two chiral centers, were found to be high-potency 5-HT_2A_ receptor agonists (EC_50_ values ≤ 1 nM).^82^ Structurally related isotryptamine analogs of **40** lacking the amide function were also shown to be 5-HT_2C_ agonists.^83^ The isotryptamine analog of LSD (i.e., **40** where X = H), with the same stereochemistry as LSD (i.e., at the LSD 5- and 8-positions), has been reported as (+)­JRT; this compound is a potent partial agonist at 5-HT_2A_ and 5-HT_2B_ receptors (*E*_max_ = 81% and 48%, respectively) but nearly a full agonist at 5-HT_2C_ receptors (*E*_max_ = 89%).^84^

**Figure 11:**
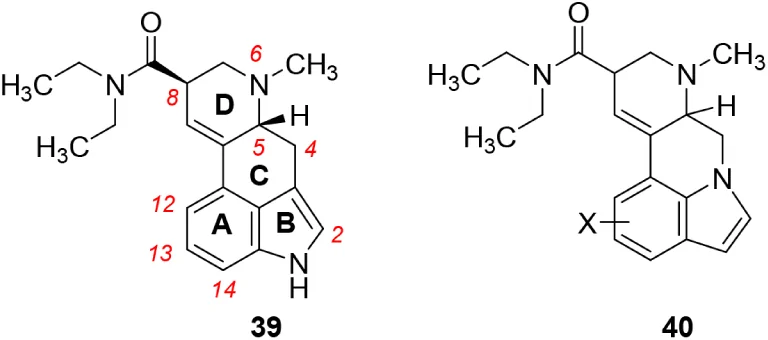
Lysergic acid diethylamide (LSD; **39**) and isotryptamine counterparts (**40**).

### Behavioral Assays

Out of necessity, hallucinogenic agents have been studied in an array of nonhuman animal behavioral assays. For example, various hallucinogenic agents have been examined in tests of stimulus generalization (i.e., substitution) in drug discrimination studies with rats trained to discriminate the classical hallucinogen/psychedelic agent and 5-HT_2_ receptor agonist 1-(2,5-dimethoxy-4-methylphenyl)-2-aminopropane (DOM; **16**) from vehicle. For a series of two dozen agents, there was a significant correlation between generalization potency and human hallucinogenic potency; subsequent studies revealed a correlation among 5-HT_2A_ receptor affinity, discrimination-derived ED_50_ values, and human hallucinogenic dose.^85^ Various “animal models of hallucinogenic” action have been described in the literature, but no animal model has proven foolproof and occasionally identifies “false positives” or “false negatives”;^86,87^ drug discrimination studies are no exception.^87^ Lisuride is a case in point. A member of the ergoline family, as is LSD (**39**), lisuride binds at 5-HT_2A_ (as well as 5-HT_2C_ and dopamine) receptors. Both are partial agonists at 5-HT_2A_ and 5-HT_2C_ receptors,^88^ but lisuride is generally considered to lack hallucinogenic character in human subjects. Nevertheless, lisuride substituted for DOM^88^ and *R*(−)­DOM,^89^ as did LSD, as well as for LSD^89,90^ in drug discrimination studies, and its effect in *R*(−)­DOM-trained animals was blocked by ketanserin and pirenperone.^89^ These might be considered false positives; however, a follow-up study revealed that a lisuride dose (i.e., 0.01 mg/kg), well below the substitution dose (i.e., 0.6 mg/kg), attenuated DOM-appropriate responding in DOM-trained animals, suggesting that lisuride might act as a weak partial agonist at 5-HT_2_ receptors.^91^ In addition, rats have been trained to discriminate LSD (0.08 mg/kg) from lisuride (0.04 mg/kg) from vehicle in a three-lever operant procedure, showing that the two agents can produce recognizably different stimulus effects.^90^ Moreover, the 5-HT_2_ antagonist pirenperone potently blocked the stimulus effects of LSD, whereas those of lisuride were blocked by the dopamine antagonist haloperidol; pirenperone also attenuated the stimulus effects of lisuride, but a 10-fold higher dose was required than that for blocking the effects of LSD.^90^ It was concluded that LSD and lisuride might produce their stimulus effects by different mechanisms.^90^ Some have attempted to explain differences in downstream 5-HT_2A_ receptor signaling efficacy between LSD and lisuride (as well as between other hallucinogenic and nonhallucinogenic agents) as the basis for why some 5-HT_2A_ receptor agonists are not hallucinogenic/psychedelic.^92,93^ Others have speculated that an increase in phosphoinositide levels above a threshold level might be required for the discriminative stimulus actions of hallucinogens.^94^ On the basis of cocrystallographic studies, LSD and lisuride bind at 5-HT_2A_ receptors at a common site, but do so in a slightly different manner.^93^ Nevertheless, despite the existence of the few false positives that have been identified, where reliable human data are available^89,91,94^ drug discrimination studies offer a method of identifying which agents produce a common stimulus effect and a means to investigate mechanisms of action.^95,96^

Coupled with the fact that the DOM stimulus is potently antagonized by the 5-HT_2_ receptor antagonists ketanserin and pirenperone, it was hypothesized that hallucinogenic agents act as 5-HT_2A_ receptor agonists or, at least, as partial agonists.^85,95,97–99^ As an aside, it might be mentioned that *R*(−)­DOB (i.e., the more potent and higher-affinity isomer of **17**)^55,100^ and racemic DOI (**18**)^101–103^ also have been used as training drugs, and their stimulus effects are blocked by pipenperone or another 5-HT_2_ antagonist. DOM-stimulus generalization to certain agents [e.g., 5-methoxy DMT (**13**), LSD (**39**)], although promiscuous binders and not 5-HT_2A_-selective agents, could also be antagonized by ketanserin, leading to the conclusion that 5-HT_2A_ agonist action might underlie their common discriminative stimulus effects.^85,98^

A few isotryptamines have been examined in stimulus generalization studies using DOM-trained rats (Figure 12). 6-Methoxy isoDMT (**12**) and 5-methoxy DMT (**13**) substituted for the DOM stimulus; although given the relatively similar 5-HT_2A_ receptor affinity of **12** and **13** (*K*_i_ = 1,040 and 600 nM, respectively), **12** was about 6-fold less potent than **13** (ED_50_ = 7.1 and 1.2 mg/kg, respectively).^3^ Both the 5-methoxy analog of isoDMT (i.e., **14**) and its corresponding DMT counterpart (i.e., 6-methoxy DMT; **15**) with lower 5-HT_2A_ receptor affinity than either **12** or **13** (*K*_i_ > 10,000 and 7,300 nM, respectively) failed to substitute at the doses examined (Figure 12). Cameron et al.^104^ measured the activity (potency and efficacy) of 5- and 6-methoxy DMT (i.e., **13** and **15**, respectively) for all known 5-HT receptors and showed that 5-methoxy DMT (**13**) is substantially more potent and more efficacious at 5-HT_2A_ and 5-HT_2C_ receptors than its 6-position geometric isomer **15**.

**Figure 12:**
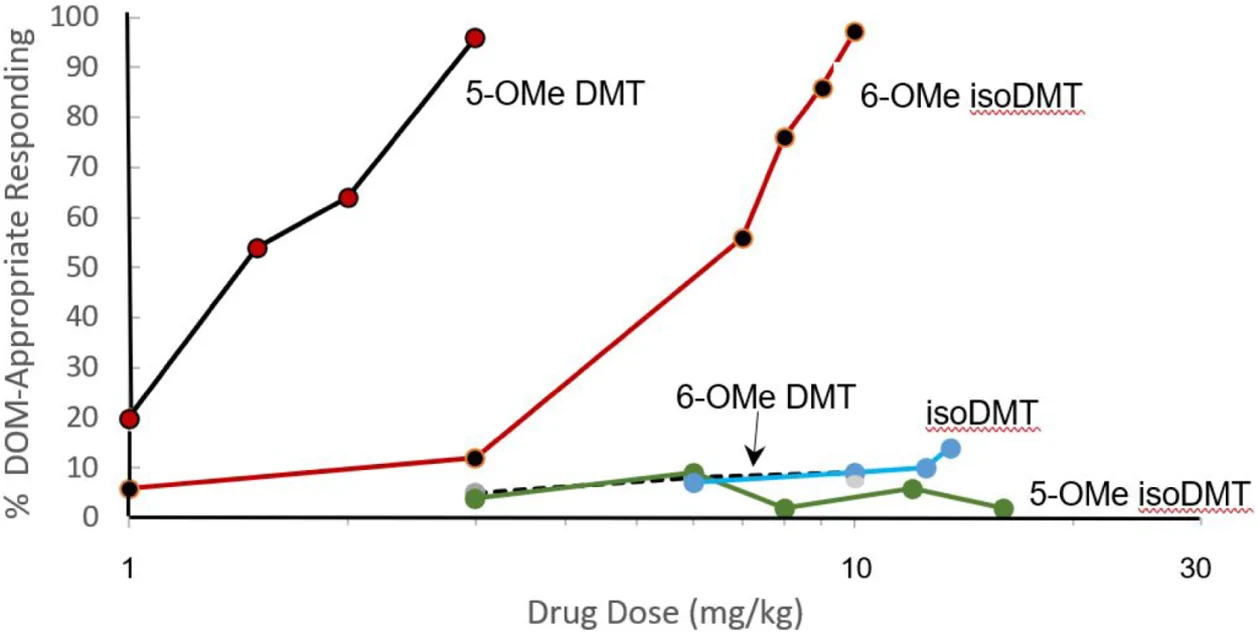
Results of stimulus generalization studies with rats trained to discriminate 1 mg/kg of DOM (**16**) from vehicle. 5-methoxy DMT (5-OMeDMT; **13**) and 6-methoxy isoDMT (6-OMe isoDMT; **12**) elicited >80% DOM-appropriate responding, whereas 6-methoxy DMT (6-OMe DMT; **15**), 5-methoxy isoDMT (5-OMe isoDMT; **14**), and isoDMT (**3**) produced vehicle-appropriate (i.e., <20% DOM-appropriate) responding. Plotted from previously published tabular data obtained using the same cohort of DOM-trained rats.^3,105^

Benzimidazole analog **11** produced 0% DOM-appropriate responding at doses of up to 12 mg/kg^3^ (data not shown). Where stimulus generalization (i.e., ≥80% DOM-appropriate responding) failed to occur in Figure 12, administration of higher drug doses disrupted the animal’s responding. DMT (**4**) also substituted in DOM-trained animals (ED_50_ = 5.8 mg/kg),^60^ whereas isoDMT (**3**) produced saline-appropriate responding (i.e., <20% DOM-appropriate responding) at doses of up to 13.5 mg/kg (Figure 12) and disruption of behavior at 15 mg/kg. Substitution, or lack thereof, might be related to differences in the pharmacokinetic properties, 5-HT_2A_ receptor efficacies, or trafficking actions of DMT versus isoDMT analogs—properties that have yet to be comparatively investigated in detail; however, evidence suggests that distribution is unlikely to play a significant role in the action of 5-methoxy DMT (**13**) and 6-methoxy isoDMT (**12**) in that, when administered (at doses that resulted in substitution) in time-course studies, both had identical durations of action.^3^

DMT (**4**) is an example of a psychoplastogen, or an agent that promotes neuronal growth, and it has been demonstrated that 5-HT_2A_ receptors are involved in its psychoplastogenic effects.^106^ Because of the neuropsychiatric potential of DMT-related compounds as therapeutic agents, a series of isoDMT analogs was investigated. Concentration–response experiments showed that certain isoDMT and DMT analogs displayed similar psychoplastogenic potencies. For example, isoDMT (**3**) and 6-methoxy isoDMT (**12**) produced maximum efficacies and had potencies comparable to their isosteric DMT counterparts (i.e., DMT, **4**, and 5-methoxy DMT, **13**).^106^ 5-Methoxy isoDMT **14** was of particular interest because it was an “*exceptional psychoplastogen, which is highly significant due to its low hallucinogenic potential in both drug-discrimination* [see Figure 12] *and head-twitch response (HTR)* [a proxy for hallucinogenic action, *vide infra*] *assays*”. Structural features of isoDMT (**3**) were examined to identify a psychoplastogen pharmacophore. Replacement of the basic amine of isoDMT (**3**) with a carbon atom, homologation of the ethyl chain to a *n*-propyl chain, conversion of the amine to an amide (i.e., replacing the α-methylene group with a carbonyl group), and introduction of a ring nitrogen atom (i.e., benzimidazole **11**) failed to promote neuronal growth.^106^

Several different unconditioned rodent behaviors also have been used as a model or proxy for potential hallucinogenic activity in humans. Perhaps the most common and widely employed today is the rodent (usually mouse) head-twitch response or HTR (sometimes referred to as head-shake behavior), first reported in 1967 by Corne and Pickering.^107^ The HTR is typically produced by hallucinogenic agents including 5-methoxy DMT (**13**), DOI (**18**), LSD (**39**), and mescaline, and, where examined, the effect is antagonized by 5-HT_2_ receptor antagonists such as ketanserin or pirenperone (reviewed in refs 108–111 ). Interestingly, even lisuride doses of up to 3.2 mg/kg failed to induce the HTR in mice; lisuride reduced baseline HTR, although the effect was not statistically significant.^110^ However, others have reported that the 5-HT_1A_ receptor agonist 8-hydroxy-2-(*N*,*N*-di-*n*-propylamino)­tetralin (8-OH DPAT) (27 μmol/kg), LSD (**39**) (<0.9 μmol/kg), and lisuride (0.1 μmol/kg), but not 5-methoxy DMT (**13**), were able to block the 5-hydroxytryptophan-induced HTR in rats.^112^ Compared to 5-methoxy DMT (**13**), which produced a robust, dose-dependent HTR that was greater in female than male mice, 6-methoxy isoDMT (**12**) was significantly less potent; neither 6-methoxy DMT (**15**) nor 5-methoxy isoDMT (**14**) produced the HTR.^106^

The zebrafish model of development has been used to test various aspects of cognitive function, including habituation, learning, memory, and attention, as well as to differentiate changes in sensorimotor function from cognition.^113^ Dunlap et al.^106^ treated zebrafish with 5-methoxy DMT (**13**), 6-methoxy isoDMT (**12**), 6-methoxy DMT (**15**), and 5-methoxy isoDMT (**14**) and found a misclassification of isosteric pairs (as previously found in receptor assays); that is, 5-methoxy DMT and 6-methoxy isoDMT, or 6-methoxy DMT and 5-methoxy isoDMT, were likely.

## Human Studies

Various tryptamines, most of which are *N*,*N*-disubstituted analogs, such as DMT (**4**), psilocybin, 5-methoxy DMT (**13**), and LSD (**39**), are currently being examined for use in treatment-resistant depression.^2,14,19,21,22,24^ They seem to produce their effects with a rapid onset following one or two doses, but their downside is that they are psychoactive substances, with both their beneficial antidepressant and undesirable psychoactive actions being mediated, at least in part, by activation of 5-HT_2_ receptors.^114^ Of value would be 5-HT_2_ agonists devoid of the latter actions. Olson and colleagues seem to have achieved this feat by examining isoDMT (**3**) and related structures.^106^ An agent to evolve from their studies is the α-methyl analog of 5-methoxy isoDMT (**14**) or, more specifically, the *R*-isomer of 5-methoxy-*N*,*N*-dimethyl-α-methylisotryptamine (initially AAZ-A-154; now termed zalsupindole; **41**)^115^ (Figure 13); see also related references by Olson and colleagues.^22,104,106^ The mechanism of action of zalsupindole appears complex (reviewed in refs 23 and 116) and might involve several receptor systems, but it is a partial agonist at recombinant 5-HT_2A_ and 5-HT_2C_ receptors (EC_50_ = 8.2 and 3.3 μM, respectively) and is a 5-HT_2B_ antagonist. Although zalsupindole is relatively selective for 5-HT_2A_ and 5-HT_2C_ receptors, it failed to produce the HTR in rodents.^117^ Phase I clinical trials have been completed, and Phase Ib trials are currently in progress. Doses of up to 360 mg showed no signs of hallucinations, dissociation, or psychotomimetic symptoms.^116,118^

**Figure 13:**
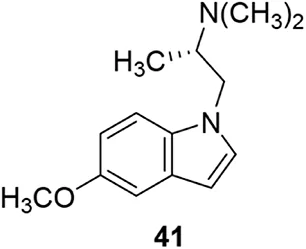
Zalsupindole (**41**).

### Related Analogs and Actions

Early studies with isotryptamines were initially focused on understanding whether and how they might bind at 5-HT receptors, employing assays available at the time. Once preliminary brain-binding data were obtained, interest turned to further investigations of their CNS pharmacology. Most studies involved analogs of isoDMT that were positional isomers of the hallucinogenic agent DMT (**4**). However, isotryptamines also serve as structural tools for the development of other types of agents (Figure 14). For example, Pazur et al.^119^ developed a series of halo-substituted 3-(2-pyridyl) analogs of isoDMT (e.g., **42**) and provided evidence as proof-of-concept for protection of light-induced retinal degeneration. To examine strictosidine synthase, an enzyme known to play a role in the biosynthesis of monoterpenoid indole alkaloids, several structurally complex and conformationally constrained strictosidine analogs (**44**) were constructed by Wu et al.,^120^ bearing a structural resemblance to **43**, where the D- and E-rings and substituents have been modified and an isotryptamine scaffold introduced.

**Figure 14:**
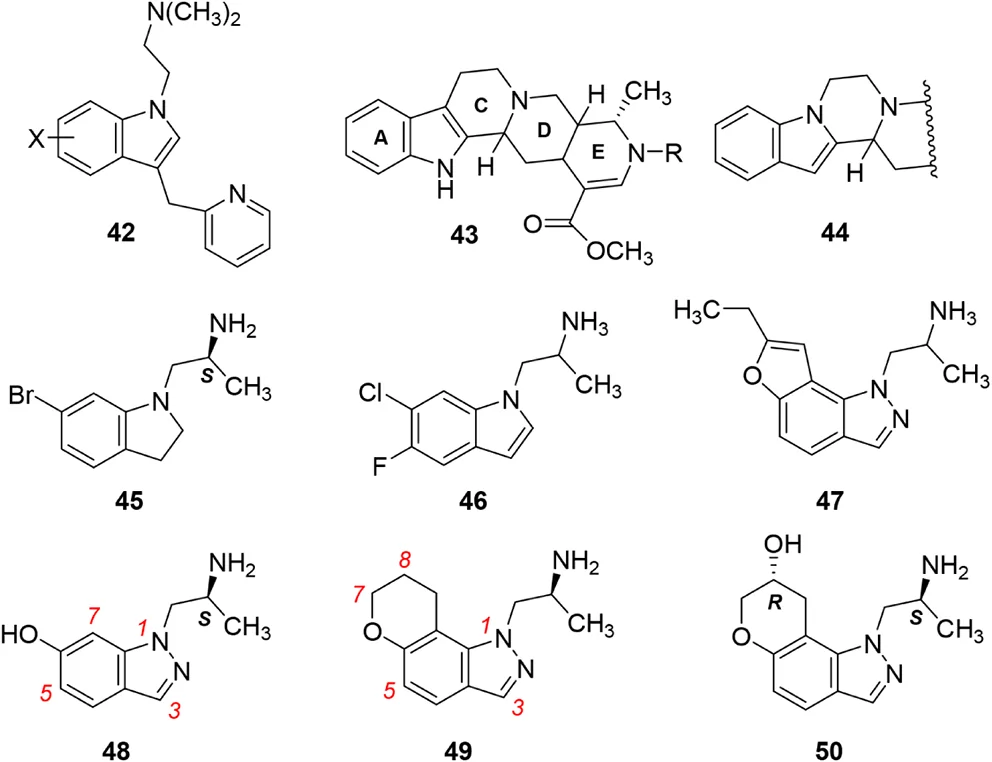
Examples of isotryptamine-related analogs with various pharmacological actions.

5-HT_2C_ receptors are thought to be involved in appetite. Due to its high affinity for human [^3^H]­5-HT-labeled 5-HT_2C_ receptors (*K*_i_ = 5.6 nM) and relative selectivity over [^125^I]­DOI-labeled human 5-HT_2A_ receptors (*K*_i_ = 355 nM), despite its affinity for 5-HT_2B_ receptors (*K*_i_ = 30 nM),^64^ the AMT-related isotryptamine (*S*)-2-[(6-bromo-2,3-dihydroindol-1-yl)]-1-(methyl)­ethylamine (VER-3323; **45**) was examined as an appetite suppressant for the treatment of obesity.^121^ For comparison, AMT (**19a**) binds with higher affinity for like-labeled 5-HT_2C_ (*K*_i_ = 2.7 nM) and 5-HT_2A_ (*K*_i_ = 16 nM) receptors.^64^ As an extension of the work conducted by Bös et al.,^63^ Shimada et al.,^122^ found that Ro60–0175 (**46**, as its *S*-isomer) binds nearly equally well at 5-HT_2C_ (*K*_i_ = 26 nM) and 5-HT_2A_ (*K*_i_ = 39 nM) receptors but is a more potent 5-HT_2C_ (EC_50_ = 52 nM; *E*_max_ = 88%) than 5-HT_2A_ (EC_50_ = 400 nM; *E*_max_ = 91%) receptor agonist (PI hydrolysis). Various isotryptamine-related compounds were prepared, including a number of indazoles such as YM348 (*S*-isomer of **47**) with a nearly 100-fold agonist potency difference as a 5-HT_2C_ (*K*_i_ = 0.89 nM; EC_50_ = 1 nM, *E*_max_ = 76%) versus 5-HT_2A_ (*K*_i_ = 13 nM; EC_50_ = 93 nM, *E*_max_ = 97%) agonist.^122^

5-HT_2_ receptor agonists, including DOI (**18**), reduce intraocular pressure (IOP) in a cynomolgus monkey model of ocular hypertension and could represent a new treatment for glaucoma. On the basis of previously reported studies with isotryptamines, a series of variously substituted indazoles was explored in an attempt to reduce their ability to penetrate the blood–brain barrier following ocular administration.^123^ Compound **48** (AL-34662) (Figure 14) was a high-affinity 5-HT_2A_ and 5-HT_2C_ agonist (Table 4), whereas its *R*(−)-enantiomer displayed lower affinity, potency, and efficacy; indazole **48** also reduced IOP in the monkey assay. Compound **48** typically displayed little affinity for most other 5-HT receptors (i.e., 5-HT_1B_, 5-HT_3_, 5-HT_5A_, 5-HT_6_; *K*_i_ > 10,000 nM), low affinity for 5-HT_4_ receptors (*K*_i_ = 7,870 nM), and reduced affinity for 5-HT_1A_ receptors (*K*_i_ = 131 nM) compared to its affinity for 5-HT_2A_ receptors (*K*_i_ = 12 nM).^123^ The *S*(+)-isomer of **48** was inactive in the mouse HTR assay at doses of up to 30 mg/kg when compared to 5-methoxy AMT (ED_50_ = 1.0 mg/kg) and the *R*(−)-isomer of DOI (**18**) (ED_50_ = 0.11 mg/kg).^123^ Introduction of a 7-methyl or 7-chloro substituent (i.e., the 7-methyl and 7-chloro analogs of **48**) resulted in compounds active in the HTR assay (ED_50_ = 0.32 and 0.56 mg/kg, respectively); in contrast, the corresponding 7-fluoro analog of **48** (i.e., the 6-hydroxy-7-fluoro analog) behaved like **48**.^123^ In tests of stimulus generalization using rats trained to discriminate DOM (**16**) (ED_50_ = 0.3 mg/kg),^124^ they failed to substitute at doses of up to 1 mg/kg and produced disruption of behavior when administered at higher doses.^124^

**Table 4:** 5-HT_2_ Receptor Affinity and Activity for Indazole Analogs **48** and **50** ^123,124^

	*K*_i_ (nM)^a^			EC_50_ (nM);^b^ Response		
	5-HT_2A_	5-HT_2B_	5-HT_2C_	5-HT_2A_	5-HT_2B_	5-HT_2C_
**48**	12	-	3.0	42.7; 99%	-	3.7; 89%
**48** epimer^c^	105	-	34	236; 61%	-	87.3; 11%
**50**	7.3	8.6	1.5	7.03; 111%	13.5; 80%	0.82; 112%

a Compound **48** and its opposite *R*-enantiomer were examined at cloned r5-HT_2_ receptors with [^125^I]­DOI as radioligand, whereas **50** was examined at cloned human 5-HT_2_ receptors using the same radioligand.

b Ca^2+^ mobilization assay using cells expressing recombinant h5-HT_2A_ receptors, rat fundus 5-HT_2B_ receptors, and cells expressing rat 5-HT_2C_ receptors.

c *R*(−)-enantiomer of **48**.

A follow-up investigation examined several constrained analogs of **48** (i.e., pyranoindazoles **49** and **50**; Tables 4 and 5).^124^ Various substituted analogs were examined; **49** (AL-38022A) (Figure 14) displayed high affinity for human 5-HT_2A_ receptors (*K*_i_ = 2.2 nM) but also displayed similar affinities for, as well as agonist action at, 5-HT_2B_ and 5-HT_2C_ receptors (Tables 4 and 5). Introduction of an 8-position hydroxyl group (i.e., **50**) resulted in slightly reduced 5-HT_2_ receptor affinity and agonist potency (Table 4).^124^ The opposite (i.e., 8-position) enantiomer of **50** showed little difference in affinity or action, suggesting that stereochemistry at this position is not important. Other than for 5-HT_1A_ receptors (IC_50_ = 687 nM), **50** displayed low affinity (IC_50_ typically ≫10 000 nM) for other 5-HT receptors. Compound **50** was effective in reducing IOP in the monkey assay and produced saline-appropriate (i.e., 12% DOM-appropriate) responding up to a dose of 0.5 mg/kg and disruption of behavior at higher doses in rats trained to discriminate DOM from vehicle.^124^

**Table 5:** Binding and Agonist Potency of AL-38022A (**49**) at 5-HT_2_ Receptor Subtypes^124^

	*K*_i_, nM^a^			EC_50_, nM;^b^ (*E*_max_ ^c^)		
	5-HT_2A_	5-HT_2B_	5-HT_2C_	5-HT_2A_	5-HT_2B_	5-HT_2C_
AL-38022A (**49**)	2.2 [0.13]	2	0.5	1.68 (113%)	3.98 (81%)	0.30 (107%)
*R*(−)DOI (*R* **18**)	0.65 [0.1]	18	4.0	0.93 (106%)	7.43 (84%)	0.87 (108%)
5-methoxy DMT (**13**)	15 [9.3]	52	42	3.4 (104%)	23.1 (75%)	9.6 (104%)
α-Me 5-HT	12 [1.0]	13	6,6	2.9 (100%)	6.1 (100%)	1.2 (106%)
5-HT	8.2 [0.3]	13	8.3	1.2 (108%)	1.6 (113%)	0.4 (100%)

a Data for [^125^I]­DOI-labeled cloned human receptors; data in brackets for 5-HT_2A_ receptors are from mice using the same radioligand.

b Based on Ca^2+^ mobilization.

c Maximal response relative to α-Me 5-HT for 5-HT_2A_ and 5-HT_2B_, and relative to 5-HT for 5-HT_2C_ receptors.

Compound **49** was likely the most thoroughly investigated isotryptamine analog until recently. It possessed a several-fold higher affinity at each of the [^125^I]­DOI-labeled human 5-HT_2_ receptor subtypes than 5-HT itself and was nearly equipotent and equi-efficacious as 5-HT as an agonist at 5-HT_2A_ and 5-HT_2C_ receptors.^124^ Table 5 provides a comparison of its actions relative to several other agents. Furthermore, **49** was quite selective for human 5-HT_2_ receptors compared to other human 5-HT receptors except for 5-HT_1A_ receptors (*K*_i_ = 294 nM), for which it still displayed about 100-fold 5-HT_2A_ selectivity; although an agonist at 5-HT_1A_ receptors, it was of very low potency (EC_50_ = 15 μM).^124^ In rats trained to discriminate DOM from vehicle, **49** (ED_50_ = 0.05 mg/kg) produced DOM-stimulus effects and was six times more potent than DOM (**16**) (ED_50_ = 0.3 mg/kg) and more potent than *R*(−)­DOI (ED_50_ = 0.2 mg/kg) and 5-methoxy DMT (**13**, ED_50_ = 0.86 mg/kg).^124^ In DOM-trained rhesus monkeys, **49** (ED_50_ = 0.04 mg/kg) was 3.5-fold more potent than DOM (ED_50_ = 0.14 mg/kg), and its actions were antagonized by the 5-HT_2A_-selective antagonist MDL-100907 (volinanserin).^124^

### Abridgment Plus

It was initially proposed that *classical hallucinogens* [i.e., those related to indolealkylamines such as DMT (**4**) and LSD (**39**), and phenylalkylamines such as mescaline, DOM (**16**), DOB (**17**), and DOI (**18**)], currently termed classical hallucinogens or classical psychedelic agents or serotonergic psychedelics, act as 5-HT_2_ (and later as 5-HT_2A_) receptor agonists in the 1980s.^97,98^ Other serotonergic mechanisms, such as 5-HT_1A_, 5-HT_1B_, and 5-HT_2C_ (initially termed 5-HT_1C_) receptor actions, might be involved in some behavioral effects of certain of these agents,^125^ but they seemed to have in common an ability to act as 5-HT_2A_ receptor agonists or partial agonists.

As new 5-HT receptor populations and subpopulations were being identified, considerable effort was devoted to identifying which 5-HT receptor (sub)­population(s) might mediate what pharmacological, physiological, or behavioral actions. An early review also intimated: “*that certain behavioral effects, thought to be mediated by a particular population of 5-HT receptors,* [might] *be modulated by a different population of 5-HT receptors is relatively novel and intriguing*”.^125^ Indolealkylamine (i.e., tryptamine) hallucinogens are notoriously nonselective 5-HT receptor agonists and often display high affinity at, for example, 5-HT_1A_ receptors. Furthermore, the 5-HT_1A_ receptor agonist 8-OH DPAT was shown to potentiate the discriminative stimulus effects of certain hallucinogenic agents used as training drugs in drug discrimination studies using DOM- and LSD-trained animals.^91,126,127^ In another study, potentiation of the DOM stimulus by 8-OH DPAT was further examined in greater detail using both of its individual optical isomers; the DOM stimulus was potentiated by racemic 8-OH DPAT and both of its optical isomers, with *R*(+)­8-OH DPAT being more potent than *S*(−)­8-OH DPAT.^128^ Administration of the ED_50_ dose of DOM in combination with a low dose of 8-OH DPAT resulted in stimulus generalization, and the potentiating effect of the racemate and *R*(+)­8-OH DPAT was blocked by the 5-HT_1A_ antagonist 1-(2-methoxyphenyl)-4-[4-(2-phthalimido)-butyl]­piperazine (NAN-190).^128^ In contrast, with a different training dose and schedule of reinforcement for DOM discrimination, 8-OH DPAT had little effect on a DOM stimulus in rats but attenuated the discriminative stimulus action of DOM in monkeys.^129^ It should also be recognized that DOM (**16**) and 5-OMe DMT (**13**), as well as LSD (**39**) and DOI (**18**),^130^ failed to substitute in rats trained to discriminate 8-OH DPAT, and that the 8-OH DPAT stimulus was not antagonized by the 5-HT_2_ receptor antagonist ketanserin.^126^ Conversely, 8-OH DPAT failed to substitute in DOM- [or *R*(−)­DOB- or DOI-] trained rats^108^ or in DOM-trained rhesus monkeys.^131^ Others have since employed tests of stimulus generalization and antagonism using 8-OH DPAT-trained rats and found that 5-methoxy DMT (**13**) and LSD (**39**) failed to substitute and that ketanserin and pirenperone failed to antagonize the stimulus effects of the training drug.^132,133^ However, Reissig et al.,^134^ using rats trained to discriminate 8-OH DPAT from vehicle, found that the 5-HT_2A_ antagonist volinanserin blocked the effect of high and intermediate doses, but not low doses, of 8-OH DPAT administration. The authors noted that various other behavioral results have also been conflicting and contradictory and that regarding drug discrimination studies, animal strain, 8-OH DPAT training dose, and schedules of reinforcement might be involved, leading them to conclude that the findings do not lend themselves to easy interpretation.^134^ It was suggested that 5-HT_2A_ and 5-HT_1A_ agonists have a bidirectional, modulatory influence upon one another in the drug discrimination paradigm.^134^ That 5-methoxy DMT failed to substitute in the 8-OH DPAT-trained animals was found “*surprising given its selectivity for the 5-HT*_*1A*_ *recepto*r”,^133^ as was the ability of lisuride, but not LSD, to substitute.^132,133^ Furthermore, the nonhallucinogenic serotonin-releasing agent fenfluramine also failed to substitute,^126^ although stimulus generalization occurred when fenfluramine was administered to DOM-trained rats.^130^ Nevertheless, it is likely that some behavioral effects of tryptamine hallucinogens are modulated due to their nonselectivity and owe some of their actions to 5-HT_1A_ (and/or some other) 5-HT receptor interactions. For example, 5-methoxy DMT (**13**) binds with higher or comparable affinity at a number of 5-HT receptors than it does at 5-HT_2A_ receptors—and only a few representative examples are provided here—human 5-HT_1A_ (*K*_i_ = 1.7 nM;^135^ 2.57 nM^136^), r5-HT_1D_ (*K*_i_ = 20 nM^137^), 5-HT_1E_ (*K*_i_ = 575 nM^19^), r5-HT_1F_ (*K*_i_ = 84 nM^137^), [^3^H]­ketanserin-labeled human 5-HT_2C_ (*K*_i_ = 77.8 nM^123^) and at rat NIH353 fibroblast cells expressing [^125^I]­DOI-labeled 5-HT_2C_ receptors (*K*_i_ = 16 nM^135^), [^125^I]­DOI-labeled human 5-HT_2C_ receptors (*K*_i_ = 0.5 nM^124^), human 5-HT_6_ (*K*_i_ = 25 nM^19^), 5-HT_7_ (*K*_i_ = 4.4 nM^19^) receptors compared to [^3^H]­ketanserin-labeled human 5-HT_2A_ receptors (*K*_i_ = 105 nM^136^) or [^125^I]­DOI-labeled rat (*K*_i_ = 42 nM^135^) or human (*K*_i_ = 15 nM^124^) 5-HT_2A_ receptors depending on receptor source or radioligand. In addition, Cameron et al.^104^ have reported that 5-methoxy DMT (**13**) is an agonist at most 5-HT receptor human isoforms [e.g., EC_50_ (nM) values/efficacy (%)]: 5-HT_1A_: 3.92/98%; 5-HT_1B_: 1.53/78%; 5-HT_1D_: 37.01/98%; 5-HT_1E_: 160.2/119%; 5-HT_1F_: 14.0/93%; 5-HT_2A_: 1.85/82%; 5-HT_2B_: 5.87/73%; 5-HT_2C_: 30.7/84%; 5-HT_5A_: 110/107%; 5-HT_6_: 0.24/125%; 5-HT_7_: 65.7/107%), but inactive at 5-HT_4_ receptors, and Blough et al.^138^ have demonstrated that 5-methoxy DMT is also a weak SERT reuptake inhibitor (IC_50_ = 2,184 nM).

A role for 5-HT_1A_ receptor involvement in the behavioral actions of tryptamines has been widely investigated and appears to be complex;^125,134^ this is not related solely to drug discrimination studies but also appears complicated regarding other behavioral models, including the HTR.^139^ 8-OH DPAT induces forepaw treading in rats (an effect common to other 5-HT_1A_ agonists, as well as fenfluramine^140^); however, following simultaneous treatment with the 5-HT_2_ agonist DOI (**18**), the potency of 8-OH DPAT was increased by about 20-fold.^141^ Although DOI did not induce forepaw treading when given alone, it induced the HTR in rats that was significantly antagonized, but not completely blocked, by 8-OH DPAT at the doses examined.^141^ The HTR in rats following administration of DOI (**18**) was blocked by various 5-HT_2_ antagonists, but the selective 5-HT_2C_ antagonist SB 200,646A failed to do so; however, 5-HT_1A_ agonists, including 8-OH DPAT, abolished the HTR.^142^ In contrast, others have found, depending on the agents examined, that 5-HT_2C_ receptor activation alone, or together with 5-HT_2A_ receptor activation, produces a comparable degree of HTR in male mice.^143^ A recent investigation also demonstrated that although 5-HT_2A_ antagonist pretreatment blocked drug-induced mouse HTR, 5-HT_1A_ antagonist pretreatment enhanced the HTR for various tryptamines, including 5-methoxy DMT (**13**), demonstrating that 5-HT_1A_ agonist action dampens the 5-HT_2A_-mediated HTR.^144^ Yet, the nonhallucinogen fenfluramine (5-HT_2A_ *K*_i_ > 5,000 nM) also produces the HTR in mice, and the selective and silent 5-HT_1A_ receptor antagonist, S-(−)­UH 301, by itself, produced the HTR in mice in a dose-dependent manner.^145^ Although DOM (**16**) binds at human 5-HT_2A_ receptors (*K*_i_ = 4.3 nM; [^125^I]­DOI) with >3,000-fold selectivity over human 5-HT_1A_ receptors (*K*_i_ = 14,200 nM; [^3^H]­8-OH DPAT),^146^ 8-OH DPAT displays modest affinity for [^3^H]­DOB-labeled r5-HT_2_ (*K*_i_ = 633 nM) receptors relative to 5-methoxy DMT (**13**; *K*_i_ = 15 nM).^147^ Why are these findings important here? Although they provide arguments for the continued use of these model systems to evaluate hallucinogen-related agents, they also provide caveats that must be considered. Even the actions of relatively selective 5-HT_2_ receptor agonists (e.g., DOM; **16**) appear to be impacted by agents that act at other 5-HT receptors. Furthermore, given that certain isotryptamines bind at receptors/transporters and protein targets in addition to 5-HT_2A_ receptors, it would seem that their actions at these receptors/targets and whether or not such interactions might modulate or augment their 5-HT_2A_-ascribed activity (action, potency) will require further investigation.

With respect to hallucinogens and 5-HT_2_ receptors, more than 40 years ago it was conjectured that “*5-HT*_2_ *agonist action might simply be a predisposing factor for such activity; hallucinogens might be 5-HT*_2_ *agonists, but will all 5-HT*_2_ *agonists be hallucinogenic?*”.^46^ How might nonhallucinogenic hallucinogens (an obvious oxymoron) or, in Naranjo’s words, “*non-psychotomimetic psychedelic agents*”,^148^ be identified? Until relatively recently, because of restrictions imposed by the Controlled Substance Act, there was a complete reliance on animal models to investigate known or potential hallucinogenic/psychedelic agents; beginning with the work of Strassman and Griffiths (see introduction), studies with human subjects have resumed. Nevertheless, various *in vitro* and *in vivo* models continue to be used. However, as stated by Box,^149^ specifically with regard to statistical models, “*all models are wrong, but some are useful*”. In 1962, Woolley, focusing primarily on serotonergic and psychoactive agents, wrote that the effects of a chemical substance on the behavior of laboratory animals are not always certain guides as to what will be the effects in humans and that the only true test is trials on patients suffering from the disease.^150^ Later, in 1968, Hollister—a clinical psychiatrist who coined the term “*chemical psychoses*” (i.e., the effect of a wide range of psychoactive or psychotomimetic agents that produce “*unusual alterations in mental function without unconsciousness*”)—considered that the effect of these agents in animals is difficult to relate to humans and is of “*dubious relevance in the··· area of behavioral pharmacology*”.^11^ These statements should be tempered somewhat considering: (i) the structural and pharmacological diversity of agents that were being initially examined (i.e., poor understanding of how *psychotomimetic* agents should be classified), (ii) the rudimentary nature of some of the animal models being used at the time—many of which were based solely on the actions of LSD, and (iii) methodological improvements in behavioral and molecular pharmacology. With respect to potential hallucinogenic drugs, the main value of animal models is to predict their actions and potency.^86^ Nearly 50 years ago, Stoff et al.,^86^ proposed ten criteria that should be met for the use of animal models to investigate hallucinogenic or psychotomimetic agents; one criterion was that “*If a specific neurotransmitter substance is indicated in the behavioral changes, then activation of that system through different mechanisms (e.g*., *receptor agonists, amine release) should yield similar effects·*·· *while inhibition through different mechanisms (e.g*., *receptor blockade) should lead to similar effects in the opposite direction*”. Despite this useful criterion, very little was known about neurotransmitter receptors and transporters at the time. Many current investigations examine the ability of an agent to produce the HTR in rodents, and some consider the results of stimulus generalization studies using rats, as indicators of possible hallucinogen action in humans. Both methods have their shortcomings,^87,108^ but a positive statistical interrelationship between results from these two models,^151^ even given some possible mechanistic differences and outliers mentioned above, strengthens arguments for their use. Moreover, as with the above-mentioned correlation between drug discrimination findings and human hallucinogenic potency, a similar correlation was found between HTR and human potency.^151^ Nevertheless, human studies should provide the most definitive information. Unfortunately, some available human findings, although perhaps predictive, are derived from reports where important details might be lacking or are anecdotal [e.g., with respect to confirmation of drug identity (particularly in self-studies found on the Internet), specific dose(s) employed, data from individual subjects, or from an unknown number of subjects]. The putative hallucinogen quipazine is a good example of this. Although quipazine, a nonselective 5-HT_2A_ receptor agonist, produces the HTR in mice and substitutes in animals (i.e., rats, monkeys) trained to discriminate hallucinogens such as DOM and LSD from vehicle, it is usually considered as a “false positive” in these models due to a paucity of reliable human data (reviewed in ref 7).

Problems exist even with documented hallucinogens; investigating the actions of various hallucinogenic agents in human subjects, Naranjo found that a given subject might react differently on different occasions of an agent’s administration.^148^ Furthermore, it is recognized that the actions of psychoactive substances can be influenced by nondrug parameters such as *set* (i.e., mind-set) and *setting* (reviewed in ref 152), and guidelines have been proposed for the clinical investigation of psychedelic agents.^153^ Documented and detailed clinical trials would seem to be required to obtain a definitive answer, but these are not conducted nowadays without preclinical data, and model systems have served well in this regard. Moreover, Szara, particularly with reference to DMT and related hallucinogens, suggested that “*Drug-induced models in animals··· could be of use as research tools if we first establish what component of the human symptom complex is reproducible in animals and then apply the result to that particular component of the symptom complex in humans*”.^154^

Another important consideration is the effect of 5-HT_2A_ receptor activation on downstream signaling. Such studies might also be viewed as models because, although they consider human data, they lack direct involvement of human subjects. It has been demonstrated that 5-HT_2A_ receptor G_q_-efficacy, as compared to 5-HT_2A_–β-arrestin2 recruitment efficacy, is necessary to produce psychedelic-like effects as measured by the mouse HTR.^155^ That is, a threshold level of G_q_ activation is required to induce psychedelic-like effects, using the HTR assay, consistent with the fact that certain 5-HT_2A_ partial agonists (e.g., lisuride) are nonpsychedelic.^155^ Although 5-HT_2A_ receptor agonists mediate activation of G_q_ and β-arrestin, there is also evidence that G_i_ signaling might be involved.^156^ Very recently, Xu et al.^1^ considered possible roles for G_i_ signaling in the actions of hallucinogenic and nonhallucinogenic agents, including isoDMT (**3**), by cryo-electron microscopy of DOI-bound (and other ligand-bound) 5-HT_2A_-G_i_ complexes. Bias between G_i_ and G_q_ signaling suggested G_q_-biased signaling is responsible for nonhallucinogenic effects. Animal models, including the HTR and marble-burying behavior—the latter considered a rodent model for anxiolytic activity—were then employed to support their findings.^1^

Overall then, *models* have been quite *useful* in that they (i) assisted in the classification of “psychotomimetic” agents as defined by Osmond^10^ and Hollister,^11^ (ii) identified a relevant neurotransmitter (i.e., 5-HT) in the common actions of classical hallucinogenic/serotonergic psychedelic agents, (iii) implicated a specific 5-HT receptor family (i.e., 5-HT_2_ receptors) that might underlie many of their actions—and it might be noted that several studies now have demonstrated that the 5-HT_2_ receptor antagonist ketanserin attenuated the subjective psychedelic effects of psilocybin,^157^ and that the general subjective effects of LSD were fully blocked by ketanserin in human subjects,^158^ (iv) provided evidence of potential involvement of other 5-HT receptors (and, indeed, other targets) in modulating the actions of these agents, and now (v) provides information on downstream signaling. It goes without saying, however, that other models are also being used. Nevertheless, prior to human evaluation, there is a need to address the actions of potential nonhallucinogenic and hallucinogenic/psychedelic agents in available model systems; perhaps even better models will be developed in the future. As stated by Vollenweider and Smallridge,^159^ “*Elucidating the biological mechanisms of classical psychedelic drugs··· persists as a promising research endeavor for neuropsychopharmacology. Realizing the full potential of psychedelic compounds as an effective and reliable clinical tool will require a continuous understanding of their interdependent effects at many biophysical and psycho-social levels*”.

## Summary

Although this is the first time that isotryptamines have been reviewed, it was not intended to be an exhaustive treatment of the topic. For example, there are various methods of isotryptamine synthesis in the chemical literature that were not discussed here, and additional isotryptamine analogs are described in some of the cited key references. Rather, this was an attempt to address the evolution and pharmacological significance of the isotryptamine chemotype, represented by isotryptamine **1**, as a novel (well, no longer so novel—given its 50-year history) scaffold for the development of agents that might be of potential value in the treatment of certain neuropsychiatric and other disorders.

Various tryptamines (as well as other agents) are currently undergoing clinical trials as possible treatments for neuropsychiatric disorders, especially depression and anxiety. This is not a novel concept; between the 1950s and mid-1960s, >1,000 scientific articles, involving 40,000 patients, were published on “*psychedelic drug therapy*”.^160^ Technological advances in a variety of fields, including the application of various model systems, have led to renewed interest. Excellent recent reviews outline the current state of psychedelic science, possible cellular mechanisms, systems neuroscience, and clinical investigation of such agents.^161,162^ A 5-HT_2_ receptor mechanism of action has been implicated for the psychoactive tryptamines, yet tryptamines are among the least selective 5-HT_2A_ receptor agonists. Involvement of other cotargets or bias in downstream signaling deserves additional scrutiny. Furthermore, because certain isotryptamines have been demonstrated to bind at 5-HT_2B_ receptors, and because this receptor population represents an antitarget for 5-HT_2B_ agonists due to its association with adverse cardiovascular events (e.g., Bender et al.^163^ and Xu et al.^164^) this is something that will require further investigation in the future. Zalsupindole (**41**) is particularly interesting because it is an isotryptamine that is a 5-HT_2A_ receptor agonist but a 5-HT_2B_ receptor antagonist.

Isotryptamines seemingly represent isosteres of tryptamines, were first described in the 1980s, and remained a curiosity for a number of years. Zalsupindole (**41**) is the first isotryptamine to undergo clinical trials and might be a revolutionary portent for the treatment of certain such disorders. Obviously, additional preclinical (i.e., “*model*”) and clinical studies with isotryptamines will be required. But, after a sluggish start, due primarily to pharmacological limitations at the time, and then scant interest in the structure type, there appears to be a recent renaissance in isotryptamine pharmacology. There is still uncertainty as to exactly how arylalkylamines (i.e., indolalkylamines such as tryptamines and phenylalkylamines such as phenylethylamines or phenylisopropylamines) produce their psychoactive effects; perhaps isotryptamines provide another structural motif to address this issue.

## Data Availability

No new data are provided here. All data have been previously reported and are cited.
